# Altered T and B lymphocyte subtype abundance in the blood of APP/PS1 mice at 6, 9 and 12 months old

**DOI:** 10.1002/alz.085155

**Published:** 2025-01-03

**Authors:** Jiaqi Sun, Yi Ling Clare Low, Xin Huang, Yoshiteru Kagawa, Colin L. Masters, Liang Jin, Yijun Pan

**Affiliations:** ^1^ University of Melbourne, Parkville, VIC Australia; ^2^ Florey Institute of Neuroscience and Mental Health, Parkville, VIC Australia; ^3^ Florey Institute of Neuroscience and Mental Health, University of Melbourne, Parkville, VIC Australia; ^4^ Monash University, Parkville, VIC Australia; ^5^ Tohoku University, Sendai Japan

## Abstract

**Background:**

Mounting evidence support the involvement of adaptive immune system in the pathogenesis of Alzheimer’s disease (AD). The current study investigated the age‐dependent changes in the abundance of B and T cell subtypes in APP/PS1 mice, a commonly used model for AD.

**Method:**

Peripheral blood was collected through cardiac puncture from 6‐, 9‐, 12‐month‐old APP/PS1 transgenic (TG) mice (APPsw^+^ and PSEN1dE9^+^, *n* = 8‐12) and their wildtype (WT) littermates (C57BL/6J, *n* = 12‐15). Flow cytometry (BD FACSCalibur™) was employed to quantify B and T lymphocytes and their subtypes. Surface markers such as CD19, Gl7, CD93, CD3, CD4, CD8 and CD25 were used for identifying early and activated B cells, as well as helper T cells, cytotoxic T cells and regulatory T cells, respectively.

**Result:**

A 3‐fold increase in the abundance of early B cells were observed in 6‐month‐old TG mice compared to WT, while their abundance decreased (42.4% *P* = 0.0106, and 46.2% *P* = 0.0431) in 9‐ and 12‐month‐old TG mice compared to WT. The abundance of activated B cells was significantly decreased in 6‐, 9‐, and 12‐month‐old TG mice compared to WT (54.3% *P* = 0.0499, 36.7% *P* = 0.0071, 45.6% *P* = 0.0082). A significant reduction in helper T cells (10.6%, *P* = 0.0056) was observed only in 6‐month‐old TG mice compared to WT. The abundance of cytotoxic T cells was found increase in 9‐ and 12‐month‐old TG mice compared to WT (1.25‐fold *P* = 0.0001, 1.20‐fold *P* = 0.0252, respectively). A significant decrease in regulatory T cells (34.5% *P* = 0.048) was noted in TG mice compared to WT at 12‐month‐old but not at earlier age.

**Conclusion:**

The study on APP/PS1 mice revealed an age‐dependent alteration in the abundance of B and T cell subtypes, supporting the involvement of adaptive immune system in AD. Future studies could address how these changes are related to AD pathological changes and confirm these changes in human blood samples.

